# Moderate Intensity Exercise in Pre-manifest Huntington’s Disease: Results of a 6 months Trial

**Published:** 2021-02-03

**Authors:** Amro Saad Aldine, Amy Ogilvie, John Wemmie, James Kent, Jordan Schultz, Jeffrey D. Long, John Kamholz, Hassan Sajjad, Joel Kline, Emily Shaw, Michelle Voss, Jane S. Paulsen, Vincent A. Magnotta

**Affiliations:** 1 Department of Radiology, Ochsner LSU Health Shreveport Academic Medical Center, Shreveport, LA, 71103, USA; 2 Department of Biostatistics, The University of Iowa, Iowa City, IA, 52240, USA; 3 Department of Psychiatry, The University of Iowa, Iowa City, IA, 52240, USA; 4 Department of Psychology, The University of Texas, Austin, Texas, 78712, USA; 5 Department of Pharmacy Practice and Science, The University of Iowa, Iowa City, IA, 52240, USA; 6 Department of Neurology, The University of Iowa, Iowa City, IA, 52240, USA; 7 Department of Internal Medicine, The University of Iowa, Iowa City, IA, 52240, USA; 8 Department of Community and Behavioral Health, The University of Iowa, Iowa City, IA, 52240, USA; 9 Department of Psychological and Brain Sciences, The University of Iowa, Iowa City, IA, 52240, USA; 10 Department of Neurology, The University of Wisconsin, Madison, WI, 53705, USA; 11 Department Radiology, The University of Iowa, Iowa City, IA, 52240, USA; 12 Department Biomedical Engineering, The University of Iowa, Iowa City, IA, 52240, USA

**Keywords:** Huntington (Disease), Neuroimaging, Moderate exercise Intensity, pre-manifest disease, MRI

## Abstract

**Background:**

While it has been shown that aerobic exercise interventions are well tolerated in participants with the Huntington disease (HD) gene mutation, no study to date has tested whether an aerobic exercise intervention benefits brain structure and function in pre-manifest HD.

**Objective:**

In this study we utilized magnetic resonance (MR) imaging techniques to assess the efficacy of moderate-to-vigorous exercise treatment relative to active stretching and toning control.

**Methods:**

Forty pre-manifest participants with confirmed HD gene expansion were recruited into a two-arm intervention study that included a moderate-to-vigorous intensity home-based walking exercise intervention (N=34) and an active stretching and toning control intervention (N=6). Participants were assessed at baseline and after 26 weeks in one of the two study arms.

**Results:**

25 of the 34 (74%) participants assigned to the moderate-to-vigorous intensity group completed the intervention while 4 of the 6 (67%) participants in the stretching and toning intervention completed the study. The primary analyses compared the two arms of the study and found no statistical differences between the groups. Both groups were found to have improved their cardiorespiratory fitness as assessed by maximal oxygen uptake (VO_2_max). A secondary analysis combined the two arms of the study and there was a significant relationship (p<0.05) between change in VO_2_max and change in brain structure.

**Conclusions:**

Though this study did not show efficacy for the exercise intervention, secondary results suggest that aerobic exercise interventions increasing cardiorespiratory fitness may be a potential way to slow progression in pre-manifest HD.

## Introduction

Huntington’s disease (HD) is a rare autosomal dominant neurodegenerative disease that occurs due to unstable expansion of the cytosine, adenine, guanine (CAG) trinucleotide repeat within the HD (*HTT*) gene on chromosome 4p16.3 [1, 2]. The disease affects primarily the central nervous system and is characterized by the progression of motor, psychiatric, and cognitive impairments [2, 3]. The age of HD diagnosis varies and is inversely related to the number of the CAG repeats within the *HTT* gene [1]. Motor manifestations of the disease usually occur around midlife, with a typical disease duration spanning 15–20 years after clinical diagnosis. To date the available treatment options provide only symptomatic relief without delaying disease onset or halting disease progression [4]. However, increasing evidence suggests that physical exercise could slow disease progression in patients with the HD gene expansion [5, 6].

A large number of neuroimaging studies have been conducted to investigate the neurodegenerative patterns induced by HD. These studies have identified a progressive volume loss specifically within the striatum [7–14]. In addition, cerebral white matter volume loss [15, 16] and micro-architectural changes [17–20] have been identified as indicators of disease progression. The neurodegenerative changes reported in HD patients are likely the result of impaired glucose metabolism [21–25] and ATP production despite normal to increased caloric intake [21, 22]. It has been suggested that mitochondrial dysfunction at the cellular level is behind the energy disturbance seen in HD [26–28]. Furthermore, lower levels of brain-derived neurotrophic factor (BDNF) have been attributed to neuronal degeneration within the striatum [29]. Most notably, studies have shown that the correction of such metabolic abnormalities may lead to a delay in disease progression [30–33].

While the number of CAG repeats within the HD gene correlates with the disease’s age of onset, the correlation is far from perfect, and there is substantial variability in the age of onset for a given CAG expansion[34]. This variance highlights the possible role of lifestyle factors such as cardiorespiratory fitness or diet in influencing the disease’s onset and progression [35]. In general, aerobic exercise, reflected in an increase in the physiological variable of cardiorespiratory fitness, has been shown to increase metabolic enzyme activity such as mitochondrial ATP synthase and glutamate dehydrogenase [36, 37], increase energy available via enhanced blood supply [38], increase central BDNF expression [39–41] and promote local glucose metabolism in the striatum [42]. In subjects without HD, moderate-to-vigorous intensity physical activity (MVPA) has been shown to improve white matter integrity [43, 44], increase caudate neuronal activity [45], and increase striatal activation during spatial learning [46]. In patients with the expanded *HTT* gene, there is increasing evidence suggesting that MVPA is well tolerated in early staged HD patients [35] and is associated with slowing symptom development [5, 6, 47–50]. However, no *in vivo* study to date has explored the effects of moderate-to-vigorous intensity aerobic exercise training on disease progression in the brains of participants with known pre-manifest HD.

In this study, the primary goal was to test the efficacy of moderate-to-vigorous exercise treatment relative to active stretching and toning control. To achieve this, we utilized magnetic resonance (MR) imaging techniques to assess the relationship between change in cardiorespiratory fitness and established markers of HD disease progression. MR imaging data collected in this study included volumetric imaging, diffusion imaging, functional connectivity, and quantitative T1 relaxation in the rotating frame (T1ρ). Recently T1ρ imaging has been shown to be associated with disease progression in pre-manifest HD patients [51]. A secondary goal was to examine these MR outcome measures to determine whether and how improvements in cardiorespiratory fitness may delay neurodegeneration in pre-manifest HD participants.

## Materials and Methods

### Participants

In our study we recruited 40 pre-manifest participants using existing observational cohorts available at the University of Iowa (PREDICT-HD: https://predict-hd.lab.uiowa.edu/, Enroll-HD: https://www.enroll-hd.org/). All participants had previously tested positive for the HD gene mutation (CAG length ≥ 36) and had a score of less than 4 on the Diagnostic Confidence Level (DCL) of the Unified Huntington’s Disease Rating Scale (UHDRS) [52] meaning that they had not yet manifested motor symptoms indicative of diagnosable HD. Our sample consisted of 15 men and 25 women whose age ranged from 20 to 60 years. Participants for this study were recruited from across the country with 42.5% outside of driving distance of the study site. See Tables 1 and 2 for additional demographic information on the participants. All participants had no history of other neurological disorders and had no contraindications for MR imaging. Participants were assessed for eligibility to participate in a moderate-to-vigorous intensity walking exercise intervention based on the Physical Activity Readiness Questionnaire [53]. In addition to that, participants were screened for the risk of an acute cardiovascular event based on the published standards of the American College of Sports Medicine (ACSM) and were qualified as “low risk”. Signed informed consent was obtained before beginning the study, per the Institutional Review Board at the University of Iowa. All experiments were performed following the guidelines outlined in the Belmont Report. Participants were assigned to one of two intervention arms with 34 assigned to the active intervention arm and 6 assigned to the control arm. Assignment to the groups was originally targeted to be a 2:1 ratio with a larger active intervention arm to better understand the barriers to completion of the intervention. However, the study failed to reach these targets due to the limited budget for the study, the higher drop rate than initially anticipated, and the greater than expected number of non-local subjects requiring additional travel costs for the study. Despite the imbalanced groups, there was no statistically significant difference between the demographics for the two cohorts. Figure (A) shows the consort diagram of progress through the study.

### Assessments

MR images were obtained using a GE 750W 3.0T scanner using a thirty-two channel head coil. Three-dimensional coronal anatomical T1-weighted BRAVO sequence (TE=3.4ms, TR=8.6ms, TI=450ms, flip angle=12°, Bandwidth=244 Hz/ pixel, Matrix=256×256×260, FOV=210×210×208mm, Acceleration=2.0 phase x 1.0 slice) and T2-weighted CUBE sequence (TE=98ms, TR=3000ms, Bandwidth=488 Hz/pixel, Matrix=256×256×260, FOV=210×210×208mm, Acceleration =2.0 phase x1.0 slice) were acquired. Resting state functional connectivity data was collected using an axial T2* weighted gradient-echo echo-planar sequence (TE=30ms, TR=2000ms, flip angle = 80°, Bandwidth=1953Hz/pixel, Matrix=64×64, FOV=220×220mm, Slice thickness/gap= 4.0 / 0.0mm, Acceleration=2.0 phase, Number of Measurements=165). Diffusion weighted images were collected in the axial plane using a spin-echo echo-planar sequence (TE=99.4ms, TR=10,675ms, bandwidth = 3906Hz/pixel, Number Diffusion Directions=30, b-value=1000s/mm^2^, Acceleration=2.0 phase) with 4 b=0 images. The phase encoding for the diffusion weighted scan was reversed to collect 2 b=0 images for use with distortion correction. Finally, 3D T1ρ images were collected using a CUBE sequence in the sagittal plane (TE=32ms, TR=3000ms, Bandwidth = 488Hz/pixel, Matrix=128×128×108, FOV=256×256×216mm, Acceleration =2.0 phase x1.0 slice, Spin-lock Frequency= 500Hz, Spin-lock durations=10 and 50ms). The total imaging time was 30 minutes.

After participants completed the MR session, their cardiorespiratory fitness was assessed using a Storer maximal bicycle test [54]. Electrocardiogram (ECG), blood pressure, and oxygen consumption were monitored during the test. Staff conducting these assessments were blinded to participant’s group assignment. Subjects were asked to maintain their pedaling between 60–100 revolutions per minute (rpm) and the resistance was increased every two minutes. The maximal exercise test was terminated when the participant was no longer able to pedal at 60 rpm for more than 15 seconds. Maximal oxygen uptake (VO_2_ max) was determined as the maximum volume of oxygen recorded when two of the following criteria were met [55]: (1) an oxygen uptake plateau between two or more workloads, (2) a respiratory exchange ratio of ≥ 1.10, and (3) a heart rate of ≥ 85% of the age predicted maximum heart rate (HRmax). A physician or licensed nurse practitioner was present to monitor participants during their maximal exercise test.

Finally, participants were also assessed using standardized measures of motor (UHDRS total motor score), function (World Health Organization Disability Assessment Schedule 2.0 Twelve Item), cognition (Symbol Digit Modalities Test, Stroop Color Naming, Stroop Word Reading, Stroop Interference, Trails Making Test), and psychiatric symptoms (Problem Behavior Assessment Short Form). These tests were administered and scored by a trained rater. This battery of assessments (MR, cardiorespiratory fitness, motor, function, cognition, and psychiatric) was completed at baseline and after completing 26 weeks of the intervention. The time period between the end of the intervention and the final assessment was 6.08 ± 4.03 days (mean ± standard deviation). Since participants were enrolled from across the United States, at home fitness activities were selected such that subjects could readily complete them in such an environment.

### Interventions

#### Moderate-to-Vigorous Intensity Intervention Arm

Participants in the aerobic exercise group underwent a moderate-to-vigorous walking intervention. Participants were given a Garmin VivoFit activity monitor with heart rate strap and instructed to undertake three brisk walking sessions per week that reached 70% of the maximum heart rate achieved during the maximal exercise test. The duration of the brisk walking exercise sessions was advanced from 15 to 50 minutes over the first 6 weeks. The program was designed to gradually build up endurance to the ACSM suggested duration 150 minutes of moderate intensity exercise per week. Participants were asked to record average heart rate and activity duration after each session in a training log that was returned to the study coordinator.

#### Active Control Arm

The active control arm consisted of a stretching and toning intervention including stretching, toning, basic core-strength, and balance exercises. These interventions are not designed to build significant muscle mass. This intervention was similar to the the balance and toning control groups that have been previously presented in the literature, which have been shown to not affect cognition [56, 57]. Participants were given a Garmin VivoFit activity monitor and instructed to complete 8–9 exercises three days a week. Each session was specified to include three lower extremity strengthening exercises, three core strengthening exercises, and two stretching exercises. In addition, they were asked to include an upper body pushup exercise during each session. Participants received this instruction from a personal trainer who also provided instructions on how to conduct the exercises. The program was designed to build up to three sets of each exercise over the first six weeks of the intervention while achieving 12–15 repetitions per set. For the stretching activities which were timed, subjects were asked to hold the position for 30 seconds. The activities for this intervention were guided based on cards from The Stretch Deck [58] and The Strength and Toning Deck [59] both from Chronicle Books (San Francisco, CA). Participants were asked to record the session activities within a training log book that was returned to the study coordinator at the end of the study.

#### Subject Monitoring

A follow-up video conference or phone call session was done within the first two weeks of the study to ensure that the subjects had found adequate routes for conducting the moderate-to-vigorous walking or if they had any questions regarding the stretching and toning exercises.

In addition, subjects were asked to upload data from the Garmin activity monitors on a weekly basis. The activity logs were reviewed and motivational messages sent via email to the subjects. These messages included the percentage of sessions that the subject had completed for the past week. Again, at the three months time point, similar motivational messages highlighting the percentage of sessions completed to date were sent to the participants.

#### Image Analysis

The anatomical T1 and T2 weighted images were processed using the FreeSurfer v6.0.0 longitudinal structural imaging pipeline [60]. This method allows for reliable extraction of volume and thickness estimates by creating an unbiased within-subject template image through inverse consistent registration [61]. The processing of the MRI scans from both time points is repeated using the within-subject template [60]. The output of the pipeline includes a tissue classified image and automated labeling (surface and volumetric labels) providing regional measurements of tissue volumes as well as cortical thickness measures. A visual representation of FreeSurfer’s automated segmentation of the caudate and putamen is shown in Figure (B).

The diffusion weighted images were visually inspected for artifacts. In addition, susceptibility-induced distortions, motion, and eddy current artifacts were corrected using the diffusion processing pipeline in FSL v6.0.0 [62]. Using this pipeline, rotationally invariant diffusion scalar measures were calculated (Fractional Anisotropy (FA), Mean Diffusivity (MD), Radial Diffusivity (RD), and Axial Diffusivity (AD)). The Mori white matter atlas was then registered to each participant using the Advanced Normalization Tools (ANTs) nonlinear registration [63]. The median rotationally invariant scalars were computed for each white matter label using tools from the BRAINStool suite (https://github.com/BRAINSia/BRAINSTools.git).

The T1ρ spin-lock images were co-registered and the resulting images were used to estimate T1ρ relaxation times using a mono-exponential fit, S_TSL_=S_0_e^−TSL/ T1ρ^ The T1ρ maps were then aligned to the anatomical T1 image output from FreeSurfer using ANTs to compute a rigid transformation. The Desikan-Killiany atlas from FreeSurfer was then used to compute median T1ρ relaxation times for each labeled brain region.

Resting state preprocessing utilized FMRIPREP v1.2.1, a Nipype based tool [64]. Each T1-weighted volume was corrected for INU (intensity non-uniformity) using N4BiasFieldCorrection v2.1.0 [65] and skull-stripped using antsBrainExtraction.sh v2.1.0 (using the OASIS template). Brain surfaces from FreeSurfer v6.0.1[66], and the brain mask estimated previously was refined with a custom variation of the method to reconcile ANTs-derived and FreeSurfer-derived segmentations of the cortical gray-matter of Mindboggle [67]. Spatial normalization to the ICBM 152 Nonlinear Asymmetrical template version 2009c [68] was performed through nonlinear registration using ANTs [69] with brain-extracted versions of both the T1-weighted volume and the ICBM template. Brain tissue segmentation of cerebrospinal fluid (CSF), white-matter (WM) and gray-matter (GM) was performed on the brain-extracted T1-weighted images using fast (FSL v5.0.9) [70].

Functional data was motion corrected using mcflirt (FSL v5.0.9) [71]. This was followed by co-registration to the corresponding T1-weighted using boundary-based registration with 9 degrees of freedom, using bbregister (FreeSurfer v6.0.1) [72]. Motion correcting transformations, BOLD-to-T1 transformation and T1-to-template Montreal Neurological Institute (MNI) warp were concatenated and applied in a single step using Lanczos interpolation. Next measures of signal flutations not thought to relate to brain activity evident in the CSF and WM were estimated from masks of each tissue type and projected to the native space of each functional run. High pass (0.008Hz) filtered regressors were created with a discrete cosine transform (DCT) basis. Frame-wise displacement was calculated for each functional run using Nipype [73]. ICA-based Automatic Removal Of Motion Artifacts (AROMA) [74] was used to generate non-aggressively denoised data.

Functional connectivity analysis was performed using atlascorr software (https://github.com/HBClab/atlascorr). The atlas used for the connectivity was created by combining atlases from Schaefer et al. [75], Choi et al. [76], and Buckner et al. [77] to provide 400, 11, and 16 functional parcels covering the entirety of the cerebral cortex, striatum, and cerebellum, respectively.

The Pearson correlation coefficient between the preprocessed time series from each parcel of interest with every other parcel was calculated for each participant and the resulting symmetric correlation matrices were converted to Fisher’s z estimates to improve normality of the outcome measure. Groupings of these brain parcels are known to share information with each other, forming networks of brain parcels corresponding to separable brain systems. Each parcel was assigned to one of 17 networks as previously identified by Yeo et al [77]. After network assignment for each parcel, the subset of parcels defining each network were extracted and put into a reduced correlation matrix defining the network of interest. For example, the Somato-Motor A network defined by Yeo [77] corresponds to execution of motor movements and the resulting correlation matrix for this network consisted of 41 parcels (39 cortical + 1 striatal + 1 cerebellar). In this study 17 networks of interest were studied as shown in supplemental Figure 1. Within each of these network matrices, we analyzed the connections between the following regions: 1) striatum with cortex, 2) cerebellum with cortex, and 3) striatum with cerebellum. Some networks did not contain parcels of interest within the striatum or cerebellum as summarized in supplemental Table 13.

#### Statistical Analysis

The primary analysis focused on the treatment effect, which involved a comparison of the two intervention arms (moderate-to-vigorous versus active control). Linear regression was used to compare 6-month brain imaging measures (dependent variable) among the groups, controlling for the pre-intervention VO_2_max, brain imaging measures, baseline age, sex, and CAG expansion. Since there was a number of subjects who did not complete the study, analyses were performed using both the participants who completed the study as well as for all participants using data imputation. Multivariate Imputation by Chained Equations (mice) was used [78] with the random forest approach. To account for the multiple tests among multiple outcome variables, the false discovery rate (FDR) correction [79] was performed separately for each imaging domain (i.e. FDR correction performed separately for the volumetric, diffusion and T1ρ data). It should be noted that the anatomical analysis normalized the region-of-interest by intracranial volume.

The primary analysis for the functional imaging data used a similar approach but analyzed the data at a network level to reduce the number of statistical comparisons performed. In this analysis, the correlations for the parcels that make up the respective broader regions of interest (i.e. cerebrum, cerebellum, and striatum) were averaged such that a single correlation coefficient represented each of the following connections: 1) striatum with cortex, 2) cerebellum with cortex, and 3) striatum with cerebellum. Using the Somato-Motor A network again as an example, this network has 39 cortical parcels resulting in 39 unique correlations with the striatum and 39 unique correlations with the cerebellum, but only one correlation between the striatum and cerebellum. The individual correlations between the cortex and either the striatum or cerebellum were averaged to ensure computational viability for imputation and to increase the reliability of the correlation measure[80]. This averaging provides an overall measure for the network strength and was used since network level change was of interest and not the parcel by parcel connectivity. Finally, a linear regression model was used to predict 6-month brain connectivity measures (dependent variable) using independent variables, which included pre-intervention VO_2_max, brain connectivity, age, sex, CAG repeat length, and group. This was done for only the study completers as well as for all subjects using data imputation as described above. The models were tested for a significant group effect after performing FDR correction.

We also conducted a secondary analysis of the data by combining the two arms of the study (moderate-to-vigorous group and active control) and used linear regression models to explore the relationship between changes in brain imaging measures and change in VO_2_max. For this analysis a change score was computed for each dependent variable (e.g. caudate volume end of study - caudate volume at baseline). The regression models included the following independent variables: change in VO_2_ max, age, sex, CAG repeat length, and baseline value for the brain imaging measure. Similar to the primary outcome analysis, FDR correction was performed separately for the volumetric, diffusion, functional connectivity, and T1ρ measurements.

## Results

Twenty-five of the 34 individuals (74%) assigned to the moderate-to-vigorous intensity exercise group completed the study while four of the six individuals (67%) assigned to the active control completed the study. Adherence data to the intervention was available in 25 of the 29 participants that completed the study. This showed that on average the moderate-to-vigorous intensity intervention arm completed 77 days of exercise (range 10–124 days) while the active control arm completed on average 73 days of the intervention (range 41–92 days). The target was 78 days for this study. Change in the cardiorespiratory fitness, symptoms, and cognition are shown in Table 3 by group. When comparing the participants that completed the study, the individuals assigned to the moderate-to-vigorous intensity intervention arm had a mean (± standard deviation) increase in VO_2_max of 1.43 ± 2.00 mL/(kg·min) while the individuals in the active control had a mean increase of 0.85 ± 2.83 mL/(kg·min). The changes in VO_2_max were statistical significant in the moderate-to-vigorous intensity intervention arm (p= 0.01) but not in the active control arm (p=0.59) using a paired t-test. It should be noted that the VO_2_max changes were not statistically different between groups as shown in Table 3. Changes on the clinical and cognitive variables showed no significant differences between groups.

The primary analysis did not identify any statistically significant differences in brain volume (Supplemental Table 1), diffusion weighted imaging (Supplemental Tables 2–3), T1ρ imaging (Supplemental Table 4), and functional connectivity (Supplemental Tables 7–8) in the moderate-to-vigorous intensity exercise group compared to the active control group. This was evident when we utilized data from not only the study completers but also all the study participants using imputation.

When we combined groups in the secondary analysis and examined the relationship between change in VO_2_max and change in imaging measures, we observed an increase in VO_2_max was significantly associated with less loss of tissue within the thalamus, pallidum, cerebellar cortex, as well as the hippocampus (Table 3) q<0.05 (using FDR correction). While the striatal changes were not statistically significant, the region did show a trend toward decreased tissue loss associated with improvements in VO_2_max (q<0.08). The slowing of tissue loss was bilateral, apart from the hippocampus where statistical significance was observed only in right hippocampus. We did not find any relationship between VO_2_max changes and diffusion tensor rotationally invariant scalars of FA and MD (Supplemental Tables 5–6). We did find a few regions (left cerebellar white matter, left caudate, and the right cerebellar cortex) where an increase in VO_2_max was associated with an increase in T1ρ relaxation times (Table 5) while an increase in VO_2_max was associated with a significant decrease in the T1ρ relaxation times within the right hippocampus (Table 5). No statistically significant relationships were observed between changes in VO_2_max and changes in functional connectivity.

## Discussion

To our knowledge, this is the first study to use MR imaging to assess the longitudinal effects of aerobic exercise in pre-manifest HD participants. The primary outcome analysis did not show any significant differences between the moderate-to-vigorous intensity intervention and active control arms in this study. However, the secondary analyses which explored the relationship in VO_2_max changes to changes in brain volume did identify a significant relationship between cardiorespiratory fitness and increased regional brain volumes. These changes were evident in multiple brain regions. While the caudate and putamen association was not statistically significant it did suggest a positive benefit of exercise on striatal volume. These findings suggest that aerobic exercise may be of potential therapeutic benefit for delaying disease progression in pre-manifest individuals with HD gene expansion. This study was not designed to assess the long -term benefits of the intervention or how training would influence the trajectory of disease progression even if adherence discontinued. A much larger study would be needed to answer these questions. This study did show that the intervention was well tolerated by the participants but that participant adherence to the intervention can be challenging.

In this study we were able to increase the VO_2_max of participants completing the walking based moderate-to-vigorous intensity intervention, which was 60% higher than the VO_2_max change in the active control arm. However, this improvement was not statistically significant. This VO_2_max change was similar in magnitude to that observed in prior exercise studies conducted on participants with idiopathic Parkinson’s Disease resulting from a three month intervention of either a high intensity treadmill intervention [81] or a similar six month home walking intervention [82]. We did have a large number of non-completers (26.47% in the moderate-to-vigorous intensity arm and 33.33% in the active control arm). This attrition rate is slightly higher than the 19% rate reported by Uc et al. in Parkinson’s Disease where subjects underwent a similar 6-month moderate-to-vigorous intensity intervention [82]. Shulman et al. reported a 11.5% drop out rate for the active arm of a high intensity treadmill intervention study lasting 3 months in Parkinson’s Disease. Similar to this study Shulman et al. observed a higher dropout rate of 21.4% in the stretching and resistance control group as compared to the active arm [81]. One potential reason for the greater dropout rate in this study as compared to these prior studies in other movement disorders is that this study was undertaken in the pre-manifest phase of the disease before the overt manifestation of symptoms. Most other exercise intervention studies in movement disorders have been undertaken after the diagnosis and participants may have noticeable improvement in their motor symptoms as a result of the exercise intervention. The improvement in motor function may have helped to keep participants engaged in the intervention. This study lacked such feedback making it relatively easy for individuals to not complete the home-based exercise intervention even with motivational messages being sent to the individuals.

In this study subjects dropped out for a variety of reasons including: 1) inability to keep up with the specified fitness activity due to other demands on the participants time (3 participants), 2) pregnancy (1 participant), 3) new development of claustrophobia (1 participant), 4) hamstring injury unrelated to fitness activity (1 participant), 4) health issue identified during baseline visit (1 participant), and 5) failure to remain engaged in the study and lost to follow-up (4 participants). Prior studies in Parkinson’s Disease have reported similar causes for participant dropout, with personal and family reasons being the most noted [81]. Future studies employing an exercise intervention in a similar pre-manifest HD populations may want to consider ways to keep individuals motivated throughout the intervention trial such as having individuals participate in group exercise routines. This could take the form of participating in spin/ aerobic classes at a local gymnasium or participating in group exercise events such as those now offered by several digital fitness applications [83].

In this study we found that greater improvements in VO_2_max were related to a slower rate of tissue loss within the hippocampus and regions involved in HD pathogenesis. This is in good agreement with the large body of literature showing that cardiorespiratory fitness is positively correlated with improved brain health manifested by increased brain volume, particularly within the gray matter of the prefrontal cortex and hippocampus [84]. In addition, cardiorespiratory fitness has been found to be associated with enhanced microstructural integrity within the white matter with a notable increase in FA measured by diffusion imaging [85–87]. For instance, cardiorespiratory fitness has been found to be correlated with an increased FA within the cingulum of adult participants [86, 87]. In Parkinson’s Disease, performance improvements in disease-specific training such as whole body dynamic balancing has been associated with time dependent increase in the right cerebellar gray matter volume [88]. Cardiorespiratory fitness in Parkinson’s Disease has also been found to be associated with an increased FA in the left putamen and caudate nucleus as well as decreased MD in the left putamen [89]. However, the current study did not find any changes in the white matter associated with changes in VO_2_max. The lack of changes in the white matter may be due to the duration of the intervention and future studies may want to consider longer interventions such as a year in length. In addition, no statistically significant results were found with functional connectivity for either the primary or exploratory analyses. Since we only observed within network correlations, we do not know if between network relationships were changed as a result of the exercise intervention.

This study has expanded upon previous aerobic exercise intervention studies [47–49] with a focus on pre-manifest HD participants. Even though we did not find significant differences between moderate-to-vigorous intensity intervention and active control arms of this study, exploratory analyses found a significant reduction in loss of gray matter as a result of improved cardiorespiratory fitness.

As grey matter volume loss can be seen as early as 10 years before the clinical manifestations [90, 91], these findings suggest the possibility that an exercise regimen may delay the onset of the disease. It should be noted however that an animal model study of preclinical HD has shown the opposite effect, that is exercise accelerating disease onset resulting in a reduced striatal volume compared to sedentary control animals [92]. In this study, the lack of a difference in the main outcome, i.e. differences between the moderate-to-vigorous intensity intervention and active control arms, may possibly be due to improved cardiorespiratory fitness observed in both groups of participants. This is not uncommon for exercise interventions and a number of prior studies have also shown increased cardiorespiratory fitness in the control arm [82]. Future work with larger sample sizes are needed to understand the long-term impact of increased cardiorespiratory fitness on the disease progression in pre-manifest HD.

One of the major limitations to this study is a high drop-out rate and the small sample size of the study. We had between 25–33% of our subjects drop out of the study within the 6-month intervention. The primary reason for dropout from the study was personal motivation and inability to keep up with the specified 50 minutes of activity three times weekly. Future exercise studies aimed at HD may want to keep this in mind and develop additional ways to increase the engagement of participants in the study. This may include activities specifically aimed to promote adherence for both experimental and comparison groups.

## Conclusion

In the participants that completed the study, we did find significant positive benefits of improved cardiorespiratory fitness that appeared to be neuroprotective. We observed significantly less loss of cortical gray matter volume associated with increased fitness. Future studies are needed to verify these findings. These findings are interesting in that they may provide a low-cost way to delay manifestation of the disease. This study also did not try to determine how long term moderate-to-vigorous exercise changes the trajectory towards HD manifestation nor does it try to describe the effects of discontinuing the exercise intervention. This would be potentially useful information to have that could further keep participants engaged in such an intervention. In addition, a broad age range was utilized in this study to increase the number of eligible subjects with this rare genetic disorder. Thus there may be different developmental and aging effects across the cohorts, which were only modeled linearly in the statistical approaches used in this study. Finally, this study was undertaken in pre-manifest HD participants where ceiling effects may exist on a number of measures including the UHDRS.

## Supplementary Material

1

## Figures and Tables

**Figure (A):**
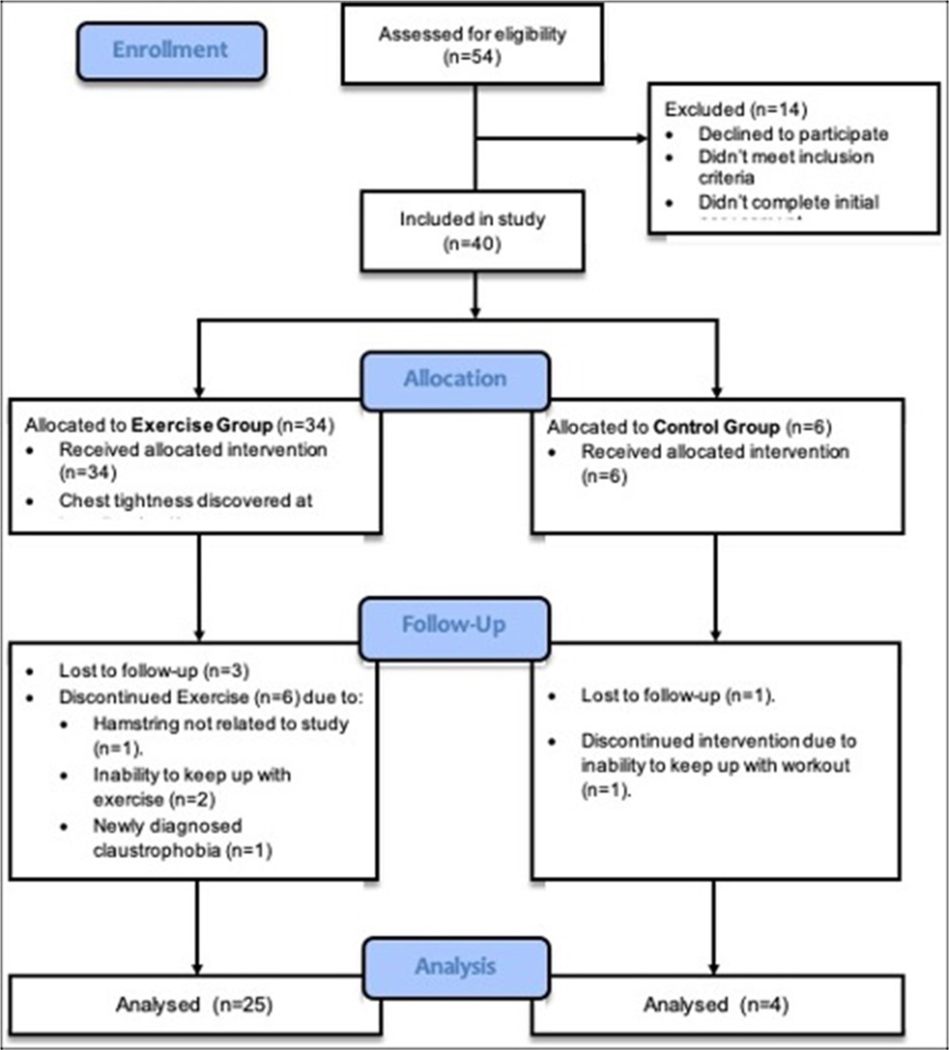
Study’s CONSORT diagram

**Figure (B):**
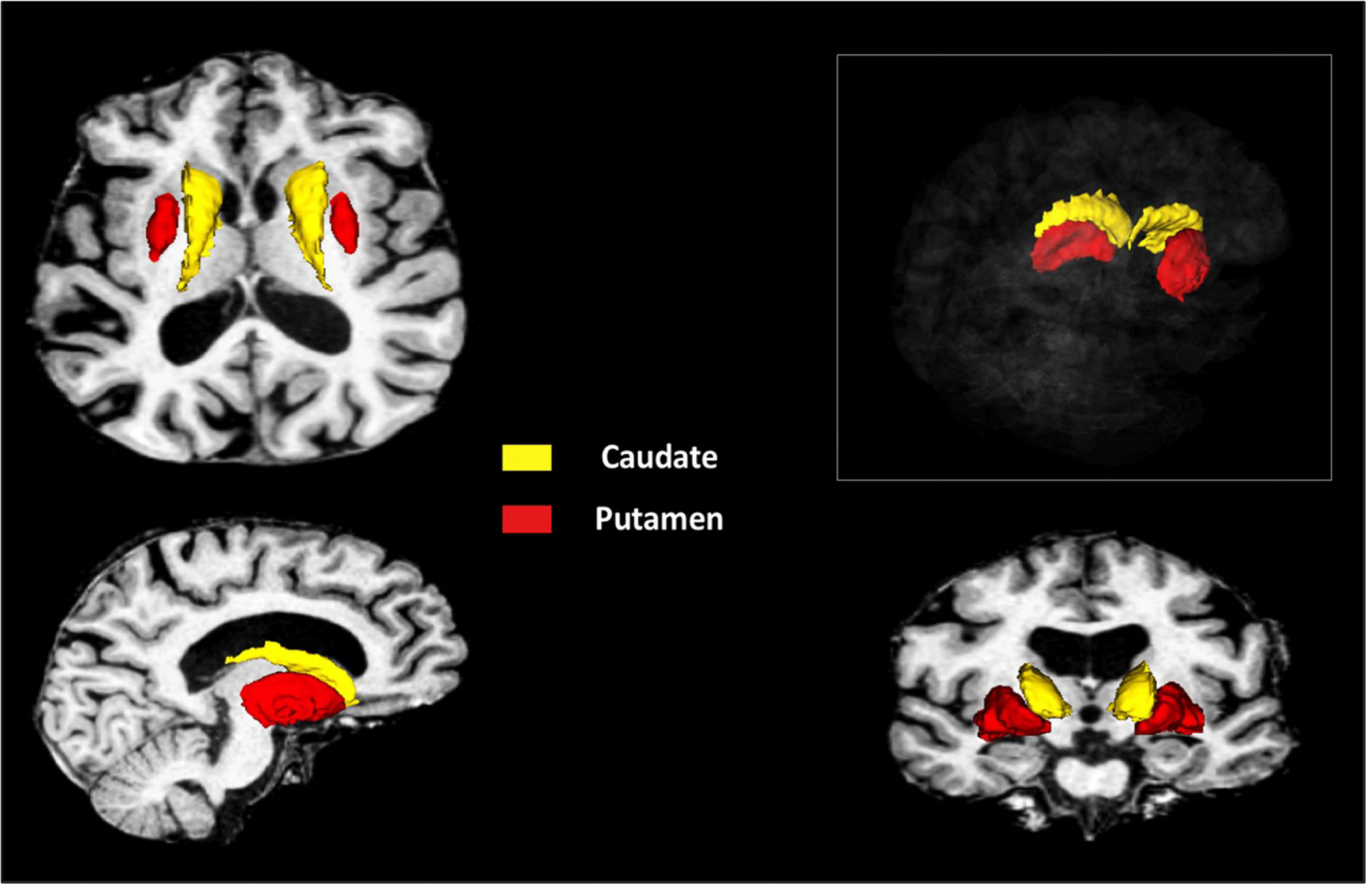
A visual representation of the caudate and the putamen volumetrically segmented using FreeSurfer

**Table 1.** Participants Demographics at Baseline for the Study*

	Overall	Moderate-to-Vigorous Intensity Intervention	Active Control	P-Value
	N = 40	N = 34	N = 6	
Sex				
Female, Count (%)	25 (62.6)	23 (67.6)	2 (33.3)	0.174
Male, Count (%)	15 (37.5)	11 (32.4)	4 (66.7)	
Age (Years)	37.08 (11.16)	37.24 (10.50)	36.17 (15.58)	0.472
BMI (kg/m^2^)	26.89 (4.20)	27.49 (4.42)	28.74 (4.47)	0.560
CAG Repeat Length	42.41 (3.23)	42.15 (2.64)	43.83 (5.67)	0.894
VO_2_ Max (mL/kg/min)	29.49 (7.51)	29.94 (7.85)	26.92 (4.83)	0.415
WHODAS***	1.71 (2.57)	1.94 (2.68)	0.20 (0.45)	0.129
UHDRS****				
Motor Score	2.38 (2.50)	2.24 (2.43)	3.17 (2.99)	0.322
Functional Score	4.93 (0.35)	4.91 (0.38)	5.00 (0.00)	0.581
SDMT*****	62.55 (10.01)	63.44 (9.77)	57.5 (10.75)	0.262
Trails Making Test				
Trails A	18.25 (6.69)	17.85 (5.99)	20.5 (10.25)	0.865
Trails B	36.48 (18.27)	35.30 (12.83)	43.17 (38.14)	0.327
Stroop Interference	54.95 (11.40)	54.29 (10.73)	58.67 (15.27)	0.881
Stroop Word Reading	106.23 (16.08)	106.85 (14.34)	102.67 (25.35)	0.327
Stroop Color Naming	85.88 (12.81)	86.26 (11.92)	83.67 (18.32)	0.928
PBA-s******				
Depression Score	1.98 (3.45)	1.94 (3.13)	2.17 (5.31)	0.638
Irritability Score	0.75 (2.13)	0.76 (2.23)	0.67 (1.63)	1.000
Apathy Score	0.325 (1.10)	0.29 (1.09)	0.5 (1.22)	0.833
Psychosis Score	0 (0)	0 (0)	0 (0)	NA
Executive Function Score	0.63 (1.66)	0.74 (1.78)	0 (0)	0.509

* Mean and (Standard deviation) are reported

** BMI=Body Mass Index

*** WHODAS= World Health Organization Disability Assessment Schedule

**** UHDRS= Unified Huntington’s Disease Rating Scale

***** SDMT= Symbol Digits Modalities Test

****** PBA-s= Problem Behavior Assessment-Short Form (Mann–Whitney U test used due to distribution shape)

**Table 2.** Demographics at Baseline for Study Completers*

	Overall	Moderate-to-Vigorous Intensity Intervention	Active Control	P-Value
	N = 29	N = 25	N = 4	
Sex				
Female, Count (%)	17 (58.6)	16 (64.0)	1 (25.0)	0.279
Male, Count (%)	12 (41.4)	9 (36.0)	3 (75.0)	
Age (Years)	39.17 (11.76)	38.72 (11.24)	42.00 (16.37)	0.849
BMI (kg/m^2^)	27.50 (4.25)	27.49 (4.42)	27.54 (3.79)	0.980
CAG Repeat Length	41.69 (2.56)	41.72 (2.69)	41.50 (1.91)	0.774
VO_2_ Max (mL/kg/min)	30.56 (7.64)	30.80 (8.09)	29.02 (4.28)	0.976
WHODAS***	1.45 (2.34)	1.64 (2.46)	0.25 (0.50)	0.375
UHDRS****				
Motor Score	2.14 (2.31)	2.16 (2.46)	2.00 (1.15)	0.672
Functional Score	4.90 (0.41)	4.88 (0.44)	5.00 (0.00)	0.614
SDMT *****	63.45 (10.03)	64.5 (9.63)	58.40 (11.52)	0.373
Trails Making Test				
Trails A	16.68 (5.38)	16.42 (5.06)	19.80 (7.59)	0.401
Trails B	32.37 (11.37)	31.13 (9.57)	36 (17.97)	0.976
Stroop Interference	54.93 (11.04)	55.5 (10.42)	52.20 (15.00)	0.471
Stroop Word Reading	107.57 (15.26)	109 (12.59)	96.2 (24.70)	0.061
Stroop Color Naming	87.03 (11.82)	87.79 (11.34)	83.4 (14.79)	0.342
PBA-s******				
Depression Score	1.07 (2.31)	1.29 (2.49)	0 (0)	0.327
Irritability Score	0.48 (1.81)	0.42 (1.84)	0.80 (1.79)	0.704
Apathy Score	0.10 (0.41)	0.13 (0.45)	0 (0)	0.795
Psychosis Score	0 (0)	0 (0)	0 (0)	NA
Executive Function Score	0.48 (1.35)	0.58 (1.47)	0 (0)	0.582

* Mean and (Standard deviation) are reported

** BMI=Body Mass Index

*** WHODAS= World Health Organization Disability Assessment Schedule

**** UHDRS= Unified Huntington’s Disease Rating Scale

***** SDMT= Symbol Digits Modalities Test

****** PBA-s= Problem Behavior Assessment-Short Form (Mann–Whitney U test used due to distribution shape)

**Table 3.** Change in Cardiorespiratory Fitness, Symptoms, and Cognition (6 month Follow up minus Baseline) *

	Overall	Moderate-to-Vigorous Intensity Intervention	Active Control	P-Value
	N = 29	N = 25	N = 4	
VO_2_ max (mL/kg/min)	1.43 (2.79)	1.52 (2.83)	0.85 (2.83)	0.681
WHODAS**	−0.10 (1.32)	−0.12 (1.42)	0.00 (0.00)	0.677
UHDRS***				
Functional Score	2.93 (0.59)	2.92 (0.64)	3.00 (0.00)	0.538
Motor Score	−0.62 (1.59)	−0.56 (1.47)	−1.00 (2.45)	0.748
SDMT****	0.10 (4.81)	0.33 (4.33)	−1 (7.25)	0.312
Trails A	−0.93 (4..08)	−0.375 (3.98)	−3.6 (3.78)	0.134
Trails B	4.14 (11.98)	2.88 (10.71)	10.2 (17.02)	0.417
Stroop Interference	2.66 (5.88)	3.25 (5.93)	−0.2 (5.26)	0.327
Stroop Word Reading	2.14 (9.12)	1.125 (7.47)	7 (15.02)	0.285
Stroop Color Naming	1.38 (8.12)	1.88 (8.35)	−1 (7.18)	0.624
PBA*****				
Depression Score	1 (2.63)	0.88 (2.47)	1.6 (3.58)	0.841
Irritability Score	−0.31 (1.89)	−0.21 (1.93)	−0.8 (1.78)	0.400
Apathy Score	0.17 (0.93)	0.04 (0.62)	0.8 (1.79)	0.582
Psychosis Score	0 (0)	0 (0)	0 (0)	NA
Executive Function Score	−0.38 (1.29)	−0.46 (1.41)	0 (0)	0.795

* Mean and (Standard deviation) are reported

** WHODAS= World Health Organization Disability Assessment Schedule

*** UHDRS= Unified Huntington’s Disease Rating Scale

**** SDMT=Symbol Digits Modalities Test

***** PBA= Problem Behavior Assessment-Short Form (Mann–Whitney U test used due to distribution shape)

**Table 4.** Results of Linear Regression Assessing the Relationship between VO_2_max Changes and Regional Brain Volume Changes Normalized to Intracranial Volume

	**Difference in Normalized Volume per unit VO_2_max	P-Value
Left Cerebellum White Matter	0.00006	0.056
Left Cerebellum Cortex	0.07097	0.033*
Left Thalamus	0.00238	0.033*
Left Caudate	0.00007	0.056
Left Putamen	0.00370	0.080
Left Pallidum	0.00063	0.033*
Left Hippocampus	0.00214	0.172
Right Cerebellum White Matter	0.00017	0.056
Right Cerebellum Cortex	0.03480	0.033*
Right Thalamus	0.00420	0.033*
Right Caudate	0.00006	0.061
Right Putamen	0.00125	0.056
Right Pallidum	0.00012	0.035*
Right Hippocampus	0.00627	0.033*

* p < 0.05

** Volumetric data normalized to intracranial volume

**Table 5.** Results of Linear Regression Assessing the Relationship between VO_2_max Changes and Regional T1rho Relaxation Times by Group

	Difference in Relaxation time per unit VO_2_ max ms/mL/(kg·min)	P-Value
Left Cerebellum White Matter	2.08483	0.030*
Left Cerebellum Cortex	1.01889	0.226
Left Thalamus	−0.02450	0.942
Left Caudate	1.87704	0.030*
Left Putamen	0.74047	0.217
Left Pallidum	0.60908	0.226
Left Hippocampus	−0.30154	0.757
Right Cerebellum White Matter	0.28909	0.812
Right Cerebellum Cortex	1.35076	0.031*
Right Thalamus	−0.52101	0.217
Right Caudate	1.05989	0.127
Right Putamen	0.60789	0.226
Right Pallidum	0.10092	0.849
Right Hippocampus	−1.58137	0.0311*

* p < 0.05
