# Supplementary material for: Moderate Intensity Exercise in Pre-manifest Huntington’s Disease: Results of a 6 months Trial
Source: SVOA Neurol. Author manuscript; Available in PMC 2022 Feb 4. (PMC8815110)
Supplement: 1 [file NIHMS1688165-supplement-1.pdf]

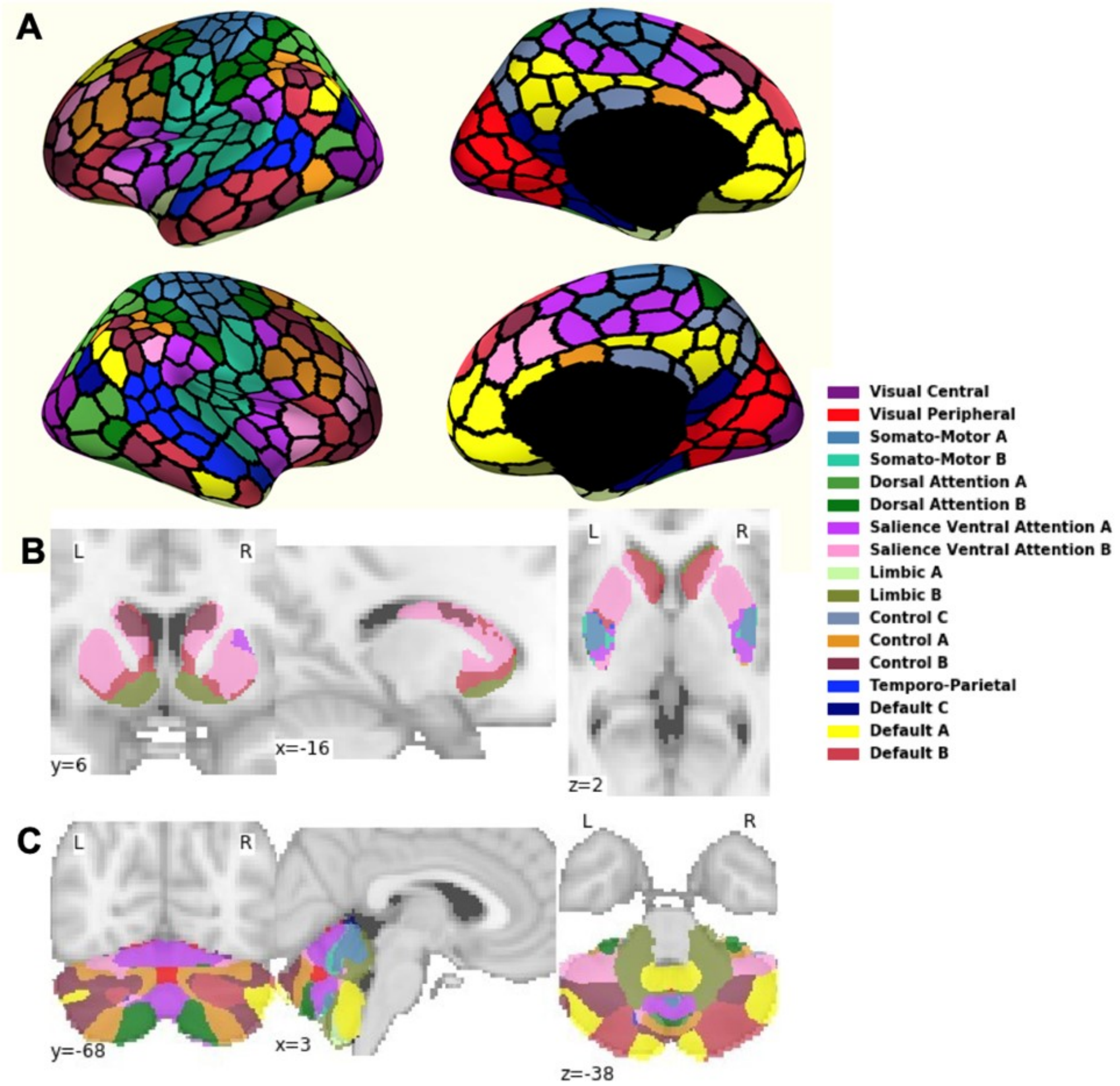

Supplemental Figure 1. *A visual representation of the parcels used for the functional connectivity analysis labeled based on network assignment. A) Shows the cortical parcels, B) the striatal parcels C) the cerebellar parcels. The regions of interest in B and C were correlated with the cortical regions of interest (A) of the same network.*

Supplemental Table 1. Normalized Tissue Volume Changes (6 month Follow-up minus Baseline) between the Moderate-to-Vigorous Intensity Intervention and Active Control Arms				
	Imputation		No Imputation	
	Difference in Volume (Moderate-to-Vigorous Intensity Intervention – Active Control)	P-Value	Difference in Volume (Moderate-to-Vigorous Intensity Intervention – Active Control)	P-Value
**Left Cerebellum White Matter**	0.00003	0.988	-0.00008	0.998
**Left Cerebellum Cortex**	0.06644	0.988	0.01615	0.998
**Left Thalamus**	0.00087	0.996	-0.00236	0.998
**Left Caudate**	-0.00014	0.988	-0.00007	0.998
**Left Putamen**	-0.00663	0.988	-0.00328	0.998
**Left Pallidum**	-0.00003	0.988	-0.00005	0.998
**Right Cerebellum White Matter**	0.00047	0.988	-0.00101	0.998
**Right Cerebellum Cortex**	0.00590	0.988	-0.01176	0.998
**Right Thalamus**	0.00068	0.988	-0.00356	0.998
**Right Caudate**	-0.00007	0.988	-0.00001	0.998
**Right Putamen**	0.00035	0.988	0.00021	0.998
**Right Pallidum**	-0.00011	0.988	0.00000	0.998

Supplemental Table 2. Rotationally Invariant Diffusion Scalar Comparison between the Moderate-to-Vigorous Intensity Intervention and Active Control Arms				
	Imputation		No Imputation	
	Difference in Fractional Anisotropy (Moderate-to-Vigorous Intensity Intervention – Active Control)	P-Value	Difference in Fractional Anisotropy (Moderate-to-Vigorous Intensity Intervention – Active Control)	P-Value
Genu of Corpus Callosum	-0.018	0.992	-0.027	0.512
Body of Corpus Callosum	-0.009	0.992	-0.009	0.711
Splenium of Corpus Callosum	-0.008	0.992	-0.010	0.742
Fornix	-0.037	0.992	-0.052	0.680
Right Corticospinal Tract	-0.002	0.992	-0.009	0.742
Left Corticospinal Tract	-0.003	0.992	-0.020	0.711
Right Anterior Limb of Internal Capsule	0.002	0.992	0.005	0.775
Left Anterior Limb of Internal Capsule	-0.007	0.992	-0.003	0.775
Right Posterior Limb of Internal Capsule	0.000	0.992	-0.002	0.872
Left Posterior Limb of Internal Capsule	-0.002	0.992	-0.006	0.775
Right Anterior Corona Radiata	-0.006	0.992	0.000	0.957
Left Anterior Corona Radiata	-0.004	0.992	-0.006	0.711
Right Superior Corona Radiata	-0.014	0.992	-0.019	0.153
Left Superior Corona Radiata	-0.002	0.992	-0.007	0.711
Right Posterior Corona Radiata	-0.008	0.992	-0.006	0.711
Left Posterior Corona Radiata	0.000	0.992	-0.004	0.775
Right External Capsule	0.008	0.992	0.011	0.711
Left External Capsule	0.001	0.992	0.633	0.775

Supplemental Table 3. Rotationally Invariant Diffusion Scalar Comparison between the Moderate-to-Vigorous Intensity Intervention and Active Control Arms				
	Imputation		No Imputation	
	Difference in Mean Diffusivity (Moderate-to-Vigorous Intensity Intervention – Active Control)	P-Value	Difference in Mean Diffusivity (Moderate-to-Vigorous Intensity Intervention – Active Control)	P-Value
Genu of Corpus Callosum	0.000	0.951	0.000	0.894
Body of Corpus Callosum	0.000	0.951	0.000	0.894
Splenium of Corpus Callosum	0.000	0.951	0.000	0.894
Fornix	0.000	0.951	0.000	0.894
Right Corticospinal Tract	0.000	0.951	0.000	0.894
Left Corticospinal Tract	0.000	0.989	0.000	0.941
Right Anterior Limb of Internal Capsule	0.000	0.951	0.000	0.894
Left Anterior Limb of Internal Capsule	0.000	0.951	0.000	0.968
Right Posterior Limb of Internal Capsule	0.000	0.951	0.000	0.939
Left Posterior limb of Internal Capsule	0.000	0.951	0.000	0.894
Right Anterior Corona Radiata	0.000	0.951	0.000	0.894
Left Anterior Corona Radiata	0.000	0.951	0.000	0.967
Right Superior Corona Radiata	0.000	0.951	0.000	0.894
Left Superior Corona Radiata	0.000	0.951	0.000	0.984
Right Posterior Corona Radiata	0.000	0.951	0.000	0.894
Left Posterior Corona Radiata	0.000	0.989	0.000	0.941
Right External Capsule	0.000	0.951	0.000	0.894
Left External Capsule	0.000	0.951	0.000	0.968

Supplemental Table 4. T1rho Relaxation Time Comparison between the Moderate-to-Vigorous Intensity Intervention and Active Control Arms of the Study

	Imputation		No Imputation	
	Difference in Relaxation Time (Moderate-to-Vigorous Intensity Intervention – Active Control) ms	P-Value	Difference in Relaxation Time (Moderate-to-Vigorous Intensity Intervention – Active Control) ms	P-Value
**Left Thalamus**	-2.120	0.909	-2.820	0.612
**Left Caudate**	-1.674	0.928	-0.835	0.983
**Left Putamen**	-3.960	0.909	-4.422	0.612
**Left Pallidum**	-3.071	0.909	-3.058	0.717
**Left Hippocampus**	-4.235	0.909	-4.454	0.612
**Right Cerebellum White Matter**	-8.186	0.909	-11.017	0.604
**Right Cerebellum Cortex**	-4.493	0.909	-6.009	0.612
**Right Thalamus**	0.370	0.969	0.053	0.983
**Right Caudate**	-2.749	0.928	-2.511	0.921
**Right Putamen**	-1.543	0.928	-2.672	0.9
**Right Pallidum**	-1.300	0.928	-1.442	0.983
**Right Hippocampus**	-1.604	0.928	-0.656	0.983

Supplemental Table 5. Results of Linear Regression Assessing the Relationship between VO_2max Changes and Regional White Matter Fractional Anisotropy		
	Difference in Fractional Anisotropy per unit VO_2 max AU/mL/(kg·min)	P-Value
Genu of Corpus Callosum	0.000	0.999
Body of Corpus Callosum	-0.002	0.999
Splenium of Corpus Callosum	0.000	0.999
Fornix	0.004	0.999
Right Corticospinal Tract	0.001	0.999
Left Corticospinal Tract	-0.001	0.999
Right Anterior Limb of Internal Capsule	0.001	0.999
Left Anterior Limb of Internal Capsule	-0.001	0.999
Right Posterior Limb of Internal Capsule	-0.001	0.999
Left Posterior Limb of Internal Capsule	0.000	0.999
Right Anterior Corona Radiata	0.000	0.999
Left Anterior Corona Radiata	-0.001	0.999
Right Superior Corona Radiata	0.000	0.999
Left Superior Corona Radiata	0.000	0.999
Right Posterior Corona Radiata	0.000	0.999
Left Posterior Corona Radiata	0.000	0.999
Right External Capsule	-0.001	0.999
Left External Capsule	-0.001	0.999

Supplemental Table 6. Results of Linear Regression Assessing the Relationship between VO_2max Changes and Regional White Matter Mean Diffusivity		
	Difference in Mean Diffusivity per unit VO_2 max $mm^2 \cdot mL/(kg \cdot min) \cdot s$	P-Value
Genu of Corpus Callosum	0	0.838
Body of Corpus Callosum	0	0.687
Splenium of Corpus Callosum	0	0.826
Fornix	0.000	0.789
Right Corticospinal Tract	0.000	0.687
Left Corticospinal Tract	0.000	0.687
Right Anterior Limb of Internal Capsule	0	0.789
Left Anterior Limb of Internal Capsule	0	0.848
Right Posterior Limb of Internal Capsule	0	0.85
Left Posterior Limb of Internal Capsule	0	0.713
Right Anterior Corona Radiata	0	0.864
Left Anterior Corona Radiata	0	0.789
Right Superior Corona Radiata	0	0.713
Left Superior Corona Radiata	0	0.789
Right Posterior Corona Radiata	0	0.789
Left Posterior Corona Radiata	0	0.713
Right External Capsule	0	0.848
Left External Capsule	0	0.96

Supplemental Table 7: Functional Connectivity between the Cerebellum and Cortical Networks Compared Between the Moderate-to-Vigorous Intensity Intervention and Active Control Arms				
**Network**	**Fisher's Z (Imputed)**	**Fisher's Z (Raw)**	**FDR P-value (Imputed)**	**FDR P-value (Raw)**
Control A	-0.043	0.018	0.920	0.959
Control B	-0.038	0.024	0.920	0.959
Control C	-0.026	-0.133	0.920	0.645
Default A	0.004	0.024	0.920	0.959
Default B	-0.040	0.012	0.920	0.977
Default C	-0.062	-0.153	0.920	0.596
Dorsal Attention A	-0.100	-0.199	0.920	0.596
Dorsal Attention B	-0.111	-0.189	0.920	0.596
Limbic A	0.003	-0.039	0.946	0.879
Limbic B	-0.025	-0.061	0.921	0.879
Salience Ventral Attention A	-0.033	-0.059	0.921	0.879
Salience Ventral Attention B	-0.048	-0.002	0.921	0.984
Somato-Motor A	-0.167	-0.177	0.921	0.596
Somato-Motor B	-0.139	-0.165	0.921	0.596
Temporo-Parietal	-0.116	-0.179	0.921	0.596
Visual-Peripheral	-0.005	-0.059	0.921	0.879

Supplemental Table 8: Functional Connectivity between the Striatum and Cortical Networks Compared Between the Moderate-to-Vigorous Intensity Intervention and Active Control Arms

Network	Fisher's Z (Imputed)	Fisher's Z (Raw)	FDR P-value (Imputed)	FDR P-value (Raw)
Control A	-0.161	-0.244	0.828	0.197
Control B	-0.086	-0.137	0.828	0.296
Default A	0.065	0.128	0.828	0.407
Default B	-0.013	-0.087	0.970	0.475
Limbic B	-0.038	-0.009	0.883	0.941
Salience Ventral Attention A	-0.005	-0.194	0.883	0.407
Salience Ventral Attention B	-0.029	-0.110	0.883	0.475
Somato-Motor A	-0.081	-0.206	0.864	0.296
Somato-Motor B	-0.137	-0.355	0.828	0.296
Temporo-Parietal	-0.055	-0.114	0.828	0.296
Visual-Central	-0.097	-0.091	0.828	0.444

Supplemental Table 9: Functional Connectivity Between the Striatum and Cerebellar Networks Compared Between the Moderate-to-Vigorous Intensity Intervention and the Active Control Arms

Network	Fisher's Z (Imputed)	Fisher's Z (Raw)	FDR P-value (Imputed)	FDR P-value (Raw)
Control A	-0.182	-0.247	0.872	0.377
Control B	-0.053	0.056	0.872	0.867
Default A	0.120	0.130	0.872	0.676
Default B	0.028	0.012	0.872	0.973
Limbic B	0.054	0.073	0.872	0.741
Salience Ventral Attention A	0.030	-0.136	0.872	0.741
Salience Ventral Attention B	0.045	0.004	0.872	0.973
Somato-Motor A	-0.041	-0.188	0.872	0.377
Somato-Motor B	-0.157	-0.426	0.872	0.170
Temporo-Parietal	-0.049	-0.201	0.872	0.377

Supplemental Table 10: Results of the Linear Regression Assessing the Relationship between VO_2max Changes and Functional Connectivity Changes between the Cerebellum and the Cerebral Cortex

Network	Fisher's r-z Correlation	FDR P-value
Control A	0.020	0.554
Control B	0.011	0.561
Control C	0.003	0.920
Default A	0.038	0.302
Default B	-0.013	0.554
Default C	0.013	0.554
Dorsal Attention A	-0.021	0.554
Dorsal Attention B	0.016	0.554
Limbic A	-0.010	0.554
Limbic B	0.030	0.302
Salience Ventral Attention A	0.021	0.554
Salience Ventral Attention B	0.017	0.554
Somato-Motor A	0.026	0.554
Somato-Motor B	0.019	0.554
Temporo-Parietal	-0.025	0.554
Vis Peri	0.001	0.968

Supplemental Table 11: Results of the Linear Regression Assessing the Relationship between VO_2max Changes and Functional Connectivity Changes between the Striatum and the Cerebral Cortex

Network	Fisher's Z	FDR P-value
Control A	0.004	0.971
Control B	-0.016	0.413
Default A	-0.020	0.413
Default B	-0.025	0.413
Limbic B	0.014	0.684
Salience Ventral Attention A	-0.001	0.976
Salience Ventral Attention B	-0.016	0.668
Somato-Motor A	-0.016	0.753
Somato-Motor B	-0.003	0.976
Temporo-Parietal	-0.026	0.413
Visual Central	0.032	0.413

Supplemental Table 12: Results of the Linear Regression Assessing the Relationship between VO_2max Changes and Functional Connectivity Changes between the Striatum and the Cerebellum

Network	Region	Fisher's Z	FDR P-value
Control A	Striatal	-0.002	1.000
Control B	Striatal	-0.025	1.000
Default A	Striatal	0.004	1.000
Default B	Striatal	-0.009	1.000
Limbic B	Striatal	0.000	1.000
Salience Ventral Attention A	Striatal	0.008	1.000
Salience Ventral Attention B	Striatal	-0.009	1.000
Somato-Motor A	Striatal	0.000	1.000
Somato-Motor B	Striatal	-0.028	1.000
Temporo-Parietal	Striatal	-0.080	0.227

Supplemental Table 13: Overview of the functional parcels/regions included in analysis

Network	Number of Cortical Parcels	Network in Cerebel-lum?	Network in Striatum?
Control A	24	yes	yes
Control B	25	yes	yes
Control C	12	yes	no
Default A	34	yes	yes
Default B	32	yes	yes
Default C	13	yes	no
Dorsal Attention A	27	yes	no
Dorsal Attention B	25	yes	no
Limbic A	13	yes	no
Limbic B	11	yes	yes
Salience Ventral Attention A	34	yes	yes
Salience Ventral Attention B	17	yes	yes
Somato-Motor A	39	yes	yes
Somato-Motor B	31	yes	yes
Temporo-Parietal	16	yes	yes
Visual-Peripheral	23	yes	no
Visual-Central	24	no	yes
